# Inhibitory Effects of Aschantin on Cytochrome P450 and Uridine 5′-diphospho-glucuronosyltransferase Enzyme Activities in Human Liver Microsomes

**DOI:** 10.3390/molecules21050554

**Published:** 2016-04-27

**Authors:** Soon-Sang Kwon, Ju-Hyun Kim, Hyeon-Uk Jeong, Yong Yeon Cho, Sei-Ryang Oh, Hye Suk Lee

**Affiliations:** 1 Drug Metabolism and Bioanalysis Laboratory, College of Pharmacy, The Catholic University of Korea, Bucheon 420-743, Korea; zuzutnseo@naver.com (S.-S.K.); jhyunkim@catholic.ac.kr (J.-H.K.); wjd1375@hanmail.net (H.-U.J.); yongyeon@catholic.ac.kr (Y.-Y.C.); 2 Natural Medicine Research Center, Korea Research Institute of Biology and Biotechnology, Chungbuk 363-883, Korea; seiryang@kribb.re.kr

**Keywords:** aschantin, human liver microsomes, time-dependent cytochrome P450 inhibition, UDP-glucuronosyltransferase, drug interaction

## Abstract

Aschantin is a bioactive neolignan found in *Magnolia flos *with antiplasmodial, Ca^2+^-antagonistic, platelet activating factor-antagonistic, and chemopreventive activities. We investigated its inhibitory effects on the activities of eight major human cytochrome P450 (CYP) and uridine 5′-diphospho-glucuronosyltransferase (UGT) enzymes of human liver microsomes to determine if mechanistic aschantin–enzyme interactions were evident. Aschantin potently inhibited CYP2C8-mediated amodiaquine *N*-de-ethylation, CYP2C9-mediated diclofenac 4′-hydroxylation, CYP2C19-mediated [*S*]-mephenytoin 4′-hydroxylation, and CYP3A4-mediated midazolam 1′-hydroxylation, with *K*_i_ values of 10.2, 3.7, 5.8, and 12.6 µM, respectively. Aschantin at 100 µM negligibly inhibited CYP1A2-mediated phenacetin *O*-de-ethylation, CYP2A6-mediated coumarin 7-hydroxylation, CYP2B6-mediated bupropion hydroxylation, and CYP2D6-mediated bufuralol 1′-hydroxylation. At 200 µM, it weakly inhibited UGT1A1-catalyzed SN-38 glucuronidation, UGT1A6-catalyzed *N*-acetylserotonin glucuronidation, and UGT1A9-catalyzed mycophenolic acid glucuronidation, with IC_50_ values of 131.7, 144.1, and 71.0 µM, respectively, but did not show inhibition against UGT1A3, UGT1A4, or UGT2B7 up to 200 µM. These *in vitro* results indicate that aschantin should be examined in terms of potential interactions with pharmacokinetic drugs *in vivo*. It exhibited potent mechanism-based inhibition of CYP2C8, CYP2C9, CYP2C19, and CYP3A4.

## 1. Introduction

**Figure 1:**
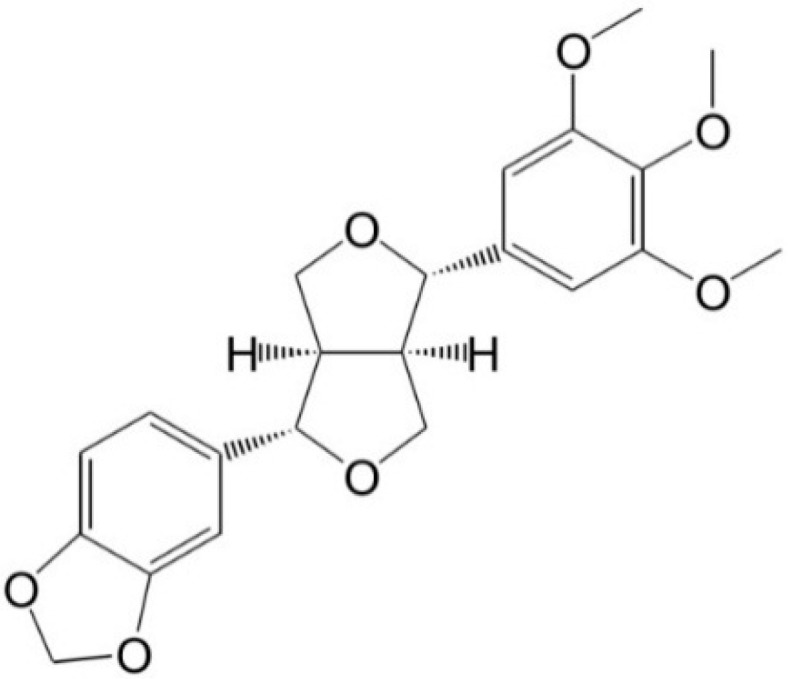
Chemical structure of aschantin.

Aschantin (Figure 1) is a bioactive neolignan found in *Magnolia* *flos* and *Hernandia nymphaeifolia* [1]. It exhibits various biological activities including peroxynitrite scavenging capacity, inhibition of inducible NO synthetase, antiplasmodial activity, Ca^2+^-antagonistic activity, platelet activating factor-antagonistic activity, and chemo-preventative or therapeutic activity mediated via inhibition of mTOR kinase [2,3,4,5,6,7].

Herbal medicines and their constituents may interact with cytochrome P450 (CYP) and uridine 5′-diphospho-glucuronosyltransferases (UGTs), increasing the possibility of herb–drug interactions [8,9,10,11,12,13]. Several medicinal herbs, including *Allium sativum*, *Camellia sinensis*, *Ginkgo biloba*, *Glycyrrhiza* *glabra*, *Coptidis* *rhizoma*, *Silybi* *fructus*, and St. John’s wort are associated with herb–drug interactions attributable to inhibition and/or induction of drug-metabolizing enzymes [14,15,16,17]. Several lignans including honokiol [18], machilin A [19], magnolol [20], schizandrins [21], schizandrol B [22], phyllantin, hypophyllantin [23], and podophyllotoxin [24] cause herb–drug interactions featuring inhibition of CYP enzymes. 

No study has yet explored the effects of aschantin on the activities of human CYP and UGT enzymes. In this study, we evaluated the inhibitory effects of aschantin on the activities of eight major human CYP enzymes (CYPs: 1A2, 2A6, 2B6, 2C8, 2C9, 2C19, 2D6, and 3A4) and UGT enzymes (UGTs 1A1, 1A3, 1A4, 1A6, 1A9, and 2B7) in pooled human liver microsomes to investigate the possibility of aschantin–drug interactions.

## 2. Results and Discussion

**Figure 2:**
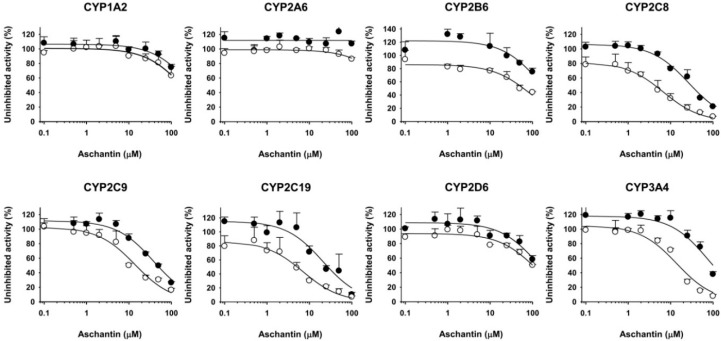
Inhibitory effects of aschantin on CYP1A2-catalyzed phenacetin *O*-de-ethylase, CYP2A6-catalyzed coumarin 7-hydroxylase, CYP2B6-catalyzed bupropion hydroxylase, CYP2C8-catalyzed amodiaquine *N*-de-ethylase, CYP2C9-catalyzed diclofenac 4′-hydroxylase, CYP2C19-catalyzed [*S*]-mephenytoin 4′-hydroxylase, CYP2D6-catalyzed bufuralol 1′-hydroxylase, and CYP3A-catalyzed midazolam 1′-hydroxylase in pooled human liver microsomes with (●) or without (○) 30 min of pre-incubation in the presence of NADPH at 37 °C. Data represent means ± standard deviations (SDs; *n* = 3).

The inhibitory effects of aschantin on eight major human CYP enzymes were evaluated in human liver microsomes (Figure 2). Aschantin moderately inhibited CYP2C8-catalyzed amodiaquine *N*-de-ethylation, CYP2C9-catalyzed diclofenac 4′-hydroxylation, CYP2C19-mediated [*S*]-mephenytoin 4′-hydroxylation, and CYP3A4-mediated midazolam 1′-hydroxylation, with IC_50_ values of 27.8, 40.5, 22.7, and 57.5 µM, respectively (Figure 2, Table 1). Aschantin at 100 µM negligibly inhibited CYP1A2-mediated phenacetin *O*-de-ethylation, CYP2A6-mediated coumarin 7-hydroxylation, CYP2B6-mediated bupropion hydroxylation, and CYP2D6-mediated bufuralol 1′-hydroxylation activities (Table 1, Figure 2). 

**Figure 3:**
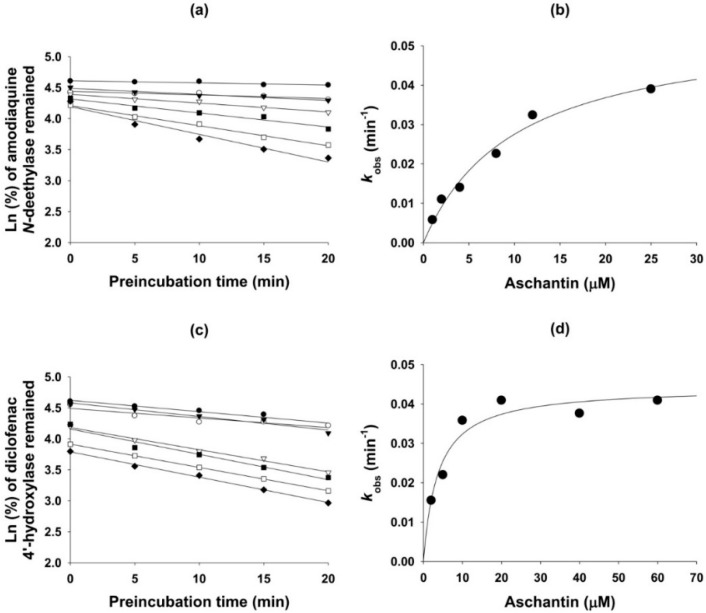
(**a**) Kinetics of inactivation of microsomal formation of *N*-de-ethylamodiaquine from amodiaquine by the following aschantin concentrations: ●, 0 µM; ○, 1 µM; ▼, 2 µM; ▽, 4 µM; ■, 8 µM; □, 12 µM; ♦, 25 µM; (**b**) The relationship between *k*_obs_ and the aschantin concentration used to estimate the *k*_inact_ and *K*_i_ values of CYP2C8-mediated amodiaquine *N*-de-ethylation; (**c**) The kinetics of inactivation of microsomal formation of 4′-hydroxydiclofenac from diclofenac by the following aschantin concentrations; ●, 0 µM; ○, 2 µM; ▼, 5 µM; ▽, 10 µM; ■, 20 µM; □, 40 µM; ♦, 60 µM, and, (**d**) the relationship between *k*_obs_ and the aschantin concentration used to estimate the *k*_inact_ and *K*_i_ values of CYP2C9-mediated diclofenac 4′-hydroxylation.

**Figure 4:**
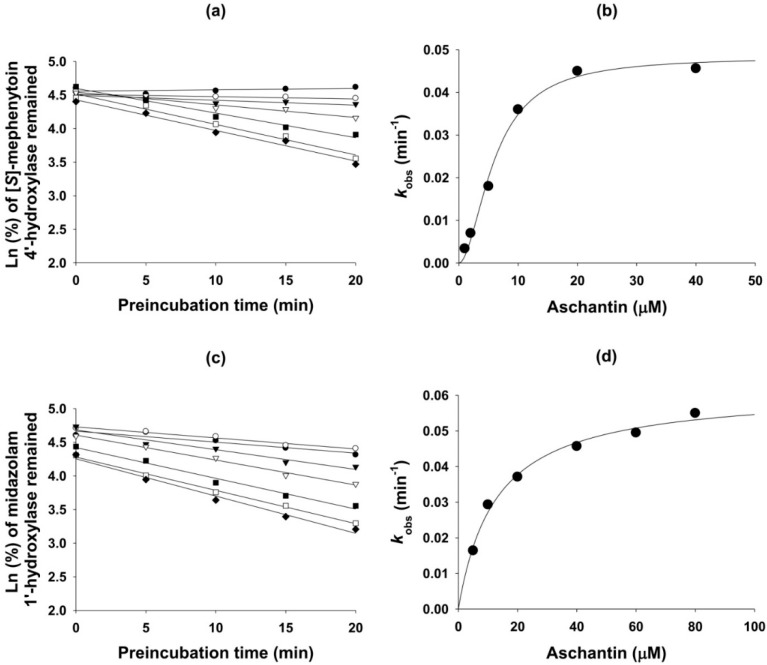
(**a**) The kinetics of inactivation of microsomal formation of 4′-hydroxy-[*S*]-mephenytoin from [*S*]-mephenytoin by the following aschantin concentrations; ●, 0 µM; ○, 1 µM; ▼, 2 µM; ▽, 5 µM; ■, 10 µM; □, 20 µM; ♦, 40 µM; (**b**) The relationship between *k*_obs_ and the aschantin concentration used to estimate the *k*_inact_ and *K*_i_ values of CYP2C19-mediated [*S*]-mephenytoin 4′-hydroxylation; (**c**) The kinetics of inactivation of microsomal formation of 1′-hydroxy midazolam from midazolam by the following aschantin concentrations; ●, 0 µM; ○, 5 µM; ▼, 10 µM; ▽, 20 µM; ■, 40 µM; □, 60 µM; ♦, 80 µM; (**d**) The relationship between *k*_obs_ and the aschantin concentration used to estimate the *k*_inact_ and *K*_i_ values of CYP3A4-mediated midazolam 1′-hydroxylation.

Aschantin lowered the IC_50_ values of CYP2C8, CYP2C9, CYP2C19, and CYP3A4 by more than 2.5-fold after 30 min pre-incubation with human liver microsomes and NADPH, compared to the values obtained after no pre-incubation (Table 1 and Figure 2), indicating that aschantin is a potent mechanism-based inhibitor of CYP2C8, CYP2C9, CYP2C19, and CYP3A4. To explore whether amodiaquine *N*-de-ethylation, diclofenac 4′-hydroxylation, [*S*]-mephenytoin 4′-hydroxylation, and/or midazolam 1′-hydroxylation were inhibited by aschantin, we performed time- and concentration-dependent inactivation assays using human liver microsomes. Aschantin decreased CYP2C8-mediated amodiaquine *N*-de-ethylation, CYP2C9-catalyzed diclofenac 4′-hydroxylation, CYP2C19-mediated [*S*]-mephenytoin 4′-hydroxylation, and CYP3A4-mediated midazolam 1′-hydroxylation, as the pre-incubation time increased, in a concentration-dependent manner (Figure 3 and Figure 4). The *k*_inact_ and apparent *K*_i_ values for aschantin were 0.056 min^−1^ and 10.2 µM for CYP2C8-mediated amodiaquine *N*-de-ethylation, 0.044 min^−1^ and 3.7 µM for CYP2C9-catalyzed diclofenac 4′-hydroxylation, 0.048 min^−1^ and 5.8 µM for CYP2C19-mediated [*S*]-mephenytoin 4′-hydroxylation, and 0.062 min^−1^ and 12.6 µM for CYP3A4-mediated midazolam 1′-hydroxylation, respectively.

**Figure 5:**
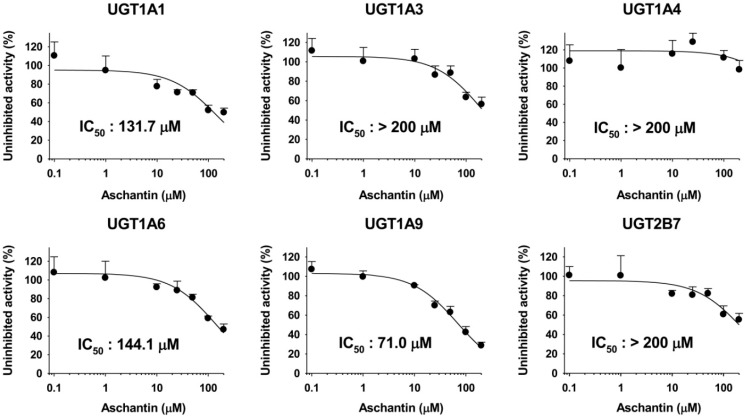
Inhibitory effects of aschantin on UGT1A1-catalyzed SN-38 glucuronidation, UGT1A3-catalyzed chenodeoxycholic acid 24-acyl-glucuronidation, UGT1A4-catalyzed trifluoperazine *N*-glucuronidation, UGT1A6-catalyzed *N*-acetylserotonin glucuronidation, UGT1A9-catalyzed mycophenolic acid glucuronidation, and UGT2B7-catalyzed naloxone 3-β-d-glucuronidationby pooled human liver microsomes. The data are means ± SDs (*n* = 3).

The inhibitory effects of aschantin on six major human UGT enzymes were evaluated using human liver microsomes (Figure 5). Aschantin weakly inhibited UGT1A1-catalyzed SN-38 glucuronidation, UGT1A6-catalyzed *N*-acetylserotonin glucuronidation, and UGT1A9-catalyzed mycophenolic acid glucuronidation, with IC_50_ values of 131.7, 144.1, and 71.0 µM, respectively. UGT1A3-catalyzed chenodeoxycholic acid 24-acyl-glucuronidation, UGT1A4-catalyzed trifluoperazine *N*-glucuronidation, and UGT2B7-catalyzed naloxone 3-β-D-glucuronidation were not inhibited by aschantin at 200 µM (Figure 5).

Aschantin was a potent mechanism-based inhibitor of CYP2C19, with a *K*_i_ value of 5.8 µM, indicating that this compound should be used carefully by patients taking CYP2C19 substrates such as amitriptyline, diazepam, imipramine, lansoprazole, omeprazole, and phenytoin, to avoid drug interactions [25]. It was also a mechanism-based inhibitor of CYP2C8, with a *K*_i_ value of 10.2 µM, indicating that aschantin should be used carefully by patients taking drugs metabolized by CYP2C8; such drugs include cerivastatin, paclitaxel, repaglinide, and sorafenib. Interactions with such drugs should be avoided [26]. 

Aschantin exhibited potent mechanism-based inhibition of CYP2C9-catalyzed diclofenac hydroxylation (*K*_i_, 3.7 µM), indicating that it should be used carefully by patients taking CYP2C9 substrates such as celecoxib, diclofenac, glyburide, losartan, tolbutamide, torasemide, and *S*-warfarin, to avoid drug interactions [27].

The compound was also a mechanism-based inhibitor of CYP3A4, with a *K*_i_ value of 12.6 µM, indicating that it should be used carefully by patients taking drugs metabolized by CYP3A4; such drugs include amlodipine, atorvastatin, cyclosporine, clarithromycin, estradiol, felodipine, lovastatin, nifedipine, ritonavir, simvastatin, and tacrolimus [25]. 

Herbal preparations containing aschantin may also exhibit time-dependent inhibition of CYP2C8, CYP2C9, CYP2C19, and CYP3A4 activities. Currently, no human data on aschantin pharmacokinetics are available; such data are essential for predicting the drug–drug interaction potential of aschantin. Our *in vitro* results suggest that aschantin should be examined in terms of potential *in vivo* pharmacokinetic drug interactions caused by inhibition of CYP2C8, CYP2C9, CYP2C19, and CYP3A4.

## 3. Experimental Section

### 3.1. Materials and Reagents 

Aschantin was isolated from dried flower buds of *Magnolia fargesii *as described previously [28] and its purity was more than 99.0%. The dried flower buds of *Magnolia fargesii *were obtained from Korea Plant Extract Bank, Korea Research Institute of Biology and Biotechnology. The pulverized materials (3 kg) were extracted for 3 days with 9 L of methanol three times and were evaporated. The methanolic extract (225 g) was suspended in 2 L of water and extracted successively with 1 L of hexane and1 L of chloroform. The chloroform soluble fraction (100 g) was subjected to silica gel column chromatography with gradient elution of hexane/acetone to yield fractions 1–19. Fraction 8 was subjected to reversed-phase column chromatography (YMCgel ODS-4, 70–230 mesh, YMC) eluting with methanol/water (7:3, *v*/*v*) to yield active fraction. The active fraction was further purified by reversed-phase high-performance liquid chromatography (Capcell PAK C18 column, 20 × 250 mm, Shiseido; methanol/acetonitrile/water (3:2:5, *v*/*v*) to give aschantin (260 mg).

Acetaminophen (catalog number, A7085), amodiaquine hydrochloride (catalog number, 1031004), coumarin (catalog number, C4261), diclofenac sodium (catalog number, D6899), 7-hydroxycoumarin (catalog number, H24003), midazolam (catalog number, 1443602), *N*-desethylamodiaquine dihydrochloride (catalog number, D039), β-nicotinamide adenine dinucleotide phosphate (reduced form) (NADPH), phenacetin (catalog number, 1513005), *N*-acetylserotonin (catalog number, A1824), alamethicin, chenodeoxycholic acid (catalog number, C9377), mycophenolic acid (catalog number, M5255), naloxone (catalog number, 1453005), naloxone 3-β-d-glucuronide (catalog number, N-099), trifluoperazine dihydrochloride (catalog number, T8516), Trizma base, and UDP-glucuronic acid (UDPGA) were purchased from Sigma-Aldrich (St. Louis, MO, USA). Pooled human liver microsomes (catalog number, 452161), bupropion (catalog number, 451740), bufuralol hydrochloride (catalog number, 451034), 1′-hydroxybufuralol maleate (catalog number, 451035), hydroxybupropion (catalog number, 451711), d_9_-1′-hydroxybufuralol maleate (catalog number, 451040), 4′-hydroxydiclofenac (catalog number, 451743), 4′-hydroxymephenytoin (catalog number, 451033), 1′-hydroxymidazolam (catalog number, 451038), and [*S*]-mephenytoin (catalog number, 451032) were obtained from Corning Life Sciences (Woburn, MA, USA). SN-38 was the product of Santa Cruz Biotechnology (Dallas, TX, USA). ^13^C_2_,^15^N-acetaminophen (catalog number, A161228), chenodeoxycholic acid-24-acyl-β-glucuronide (catalog number, C291910), mycophenolic acid-β-d-glucuronide (catalog number, M831520), and SN-38 glucuronide (catalog number, S589980) were obtained from Toronto Research Chemicals (Toronto, ON, Canada). Acetonitrile and methanol (HPLC grade) were obtained from Burdick & Jackson Inc. (Muskegon, MI, USA); all other chemicals were of the highest quality available.

### 3.2. Inhibitory Effects of Aschantin on the Activities of Seven Major CYPs in Human Liver Microsomes

The degrees of inhibition (the IC_50_ values) of aschantin toward CYP1A2, CYP2A6, CYP2C8, CYP2C9, CYP2C19, CYP2D6, and CYP3A4 were evaluated using pooled human liver microsomes employing a cocktail of CYP substrates and liquid chromatography-tandem mass spectrometry (LC-MS/MS). The incubation mixtures were prepared in total volumes of 100 µL, as follows: pooled human liver microsomes (0.2 mg/mL), 1.0 mM NADPH, 10 mM MgCl_2_, 50 mM potassium phosphate buffer (pH 7.4), various concentrations of aschantin in DMSO (final concentrations of 0.1–100 µM, DMSO less than 1% [*v*/*v*]), and a cocktail of seven CYP probe substrates as defined in our previous report [18]. The CYP substrates were used at concentrations approximating their respective *K*_m_ values: 50 µM phenacetin, 2.5 µM coumarin, 2.0 µM amodiaquine, 10 µM diclofenac, 100 µM [*S*]-mephenytoin, 5 µM bufuralol, and 2.5 µM midazolam. After 3 min pre-incubation at 37 °C, the reactions were initiated by addition of NADPH and incubation proceeded for 15 min at 37 °C in a shaking water bath. The reaction was stopped by placing the tubes on ice and adding 100 µL amounts of ice-cold methanol containing internal standards (^13^C_2_,^15^N-acetaminophen for acetaminophen and *N*-desethylamodiaquine; d_9_-1′-hydroxybufuralol for 4′-hydroxydiclofenac; and 7-hydroxycoumarin, 4′-hydroxy-mephenytoin, 1′-hydroxybufuralol, and 1′-hydroxymidazolam). Then the incubation mixtures were centrifuged at 13,000× *g* for 4 min at 4 °C. All assays were performed in triplicate and the average values were used in calculations. To measure the mechanism-based inhibition of CYP activities, various concentrations of aschantin (0.1–100 µM) were pre-incubated for 30 min with human liver microsomes in the presence of NADPH. Each reaction was initiated by adding the seven-CYP probe substrate cocktail. 

The metabolites formed from the seven substrates were simultaneously quantified using our previously described LC-MS/MS method [18]. To this end, we employed a tandem mass spectrometer (TSQ Quantum Access, Thermo Scientific, San Jose, CA, USA) coupled to a Nanospace SI-2 LC system (Shiseido, Tokyo, Japan). The column and autosampler temperatures were 50 °C and 6 °C, respectively. The mass spectrometer was equipped with an electrospray ionization (ESI) source and was operated in positive ion mode. The ESI source settings for metabolite ionization were as follows: capillary voltage, 4200 V; vaporizer temperature, 350 °C; capillary temperature 330 °C; sheath gas pressure, 35 psi; and auxiliary gas pressure, 15 psi. Quantification was performed by selected reaction monitoring (SRM) of the [M + H]^+^ ion and the related product ion for each metabolite: acetaminophen, 152.1 > 110.3; *N*-desethylamodiaquine, 328.1 > 283.0; 7-hydroxycoumarin, 163.0 > 107.2; 4′-hydroxydiclofenac, 312.0 > 231.1; 4′-hydroxy-mephenytoin, 235.1 > 150.1; 1′-hydroxybufuralol, 278.1 > 186.1, 1′-hydroxymidazolam, 341.9 > 324.0; ^13^C_2_,^15^N-acetaminophen 155.1 > 111.2; and d_9_-1′-hydroxybufuralol, 287.2 > 187.0. Analytical data were processed using Xcalibur^®^ software (Thermo Scientific). 

### 3.3. Inhibitory Effects of Aschantin on CYP2B6 in Human Liver Microsomes

The effects of aschantin on CYP2B6-catalyzed bupropion hydroxylase activity were evaluated using LC-MS/MS with pooled human liver microsomes [18]. Each incubation mixture was prepared in a total volume of 100 µL, including 1.0 mM NADPH, 10 mM MgCl_2_, 50 mM potassium phosphate buffer (pH 7.4), various concentrations of aschantin (0.1–100 µM), pooled human liver microsomes (0.2 mg/mL), and a CYP2B6-selective substrate (50 µM bupropion), as reported previously [18]. After 3 min of pre-incubation at 37 °C, each reaction was initiated by adding NADPH; incubation proceeded for 15 min at 37 °C in a shaking water bath. The reaction was stopped by placing the tubes on ice and adding 100 µL amounts of ice-cold d_9_-1′-hydroxybufuralol (the internal standard) in methanol. Then the incubation mixtures were centrifuged at 13,000× *g* for 4 min at 4 °C. All incubations were performed in triplicate, and the average values were used in calculations. To evaluate NADPH-dependent mechanism-based inhibition, various concentrations of aschantin (0.1–100 µM) were pre-incubated for 30 min with pooled human liver microsomes in the presence of NADPH. The reaction was initiated by adding bupropion. Hydroxybupropion levels were determined using the LC-MS/MS method described above; the SRM parameters were 256.1 > 238.0 for hydroxybupropion and 287.2 > 187.0 for d_9_-1′-hydroxybufuralol.

### 3.4. Inhibitory Effects of Aschantin on the Activities of Six Major UGTs in Human Liver Microsomes

The extents of aschantin on UGT1A1, UGT1A3, UGT1A4, UGT1A6, UGT1A9, and UGT2B7 were evaluated by LC-MS/MS, using pooled human liver microsomes incubated with the cocktail of UGT substrates. The method was modified from that of Joo *et al.* [29]. Each incubation mixture was prepared in a final volume of 100 µL, as follows: pooled human liver microsomes (0.2 mg/mL), 5 mM UDPGA, 10 mM MgCl_2_, 50 mM Tris buffer (pH 7.4), various concentrations of aschantin in DMSO (final concentrations of 0.1–200 µM, DMSO less than 1% [*v*/*v*]), and the UGT enzyme-specific substrate of the cocktail set (A set: 0.5 µM SN-38, 2 µM chenodeoxycholic acid, and 0.5 µM trifluoperazine; B set: 1 µM *N*-acetylserotonin, 0.2 µM mycophenolic acid, and 1 µM naloxone). After 3 min of pre-incubation at 37 °C, the reactions were initiated by addition of UDPGA; incubation continued for 60 min at 37 °C in a shaking water bath. The reaction was stopped by placing the tubes on ice and adding 50 µL ice-cold acetonitrile containing internal standards (propofol glucuronide for chenodeoxycholic acid 24-acyl-β-glucuronide and mycophenolic acid glucuronide, and meloxicam for SN-38 glucuronide, trifluoperazine glucuronide, *N*-acetylserotonin β-d-glucuronide, and naloxone 3-β-d-glucuronide). The incubation mixtures were centrifuged at 13,000× *g* for 4 min at 4 °C. All assays were performed in triplicate and the average values were used in calculations.

The metabolites formed from the six UGT cocktail substrates were simultaneously measured using the LC-MS/MS method. A tandem mass spectrometer (TSQ Quantum Access) coupled to a Nanospace SI-2 LC system was used. The column and autosampler temperatures were 50 °C and 6 °C, respectively. The mass spectrometer was equipped with an ESI source and was operated in both positive and negative ion modes. The ESI source settings for metabolite ionization were as follows: capillary voltage, 4200 V; vaporizer temperature, 350 °C; capillary temperature 330 °C; sheath gas pressure, 35 psi; and auxiliary gas pressure, 15 psi. Each metabolite was quantified via SRM in the negative ion (chenodeoxycholic acid 24-acyl-β-glucuronide, 567.2 > 391.0; mycophenolic acid glucuronide, 495.2 > 318.9; propofol glucuronide, 353.3 > 177.1) and positive ion (SN-38 glucuronide, 569.0 > 393.0; trifluoperazine glucuronide, 584.2 > 408.1; *N*-acetylserotonin-β-d-glucuronide, 395.2 > 219.0; naloxone 3-β-d-glucuronide, 504.0 > 310.0; meloxicam, 352.0 > 115.1) modes. Analytical data were processed using Xcalibur^®^ software (Thermo Scientific).

### 3.5. Mechanism-Based Inhibition of CYP Activities by Aschantin

The mechanism-based inhibitory effects of aschantin on CYP2C8, CYP2C9, CYP2C19, and CYP3A4 activities were further evaluated in human liver microsomes. The microsomes (1 mg/mL) were pre-incubated with various concentrations of aschantin in 50 mM potassium phosphate buffer (pH 7.4) in the presence of NADPH. Aliquots (10 µL) of the incubated mixtures were withdrawn at 5, 10, 15, and 20 min after incubation commenced and added to other tubes containing the CYP substrates (2 µM amodiaquine for CYP2C8, 10 µM diclofenac for CYP2C9, 100 µM [*S*]-mephenytoin for CYP2C19, or 2 µM midazolam for CYP3A4), 1 mM NADPH, 50 mM potassium phosphate buffer (pH 7.4), and 10 mM MgCl_2_, in 90 µL reaction mixtures. The second reaction was terminated after incubation for 10 min by adding 100 µL amounts of ice-cold methanol containing d_9_-1′-hydroxybufuralol. The incubation mixtures were centrifuged at 13,000× *g* for 4 min at 4 °C, and then 50 µL of each supernatant was diluted with 50 µL of water. Aliquots (5 µL) of the diluted supernatants were analyzed by LC-MS/MS, as described above. 

### 3.6. Data analysis

IC_50_ values (*i.e*., the concentrations of inhibitors required for 50% inhibition of the original enzyme activity) were calculated using Sigma Plot version 11.0 (Systat Software, Inc., San Jose, CA, USA). The apparent kinetic inhibitory potentials (the *K*_i_ values) were estimated from the fitted curves using Enzyme Kinetics version 1.1 (Systat Software Inc.).

## 4. Conclusions

Aschantin exhibited potent mechanism-based inhibition of CYP2C8-mediated amodiaquine *N*-de-ethylation, CYP2C9-mediated diclofenac 4′-hydroxylation, CYP2C19-mediated [*S*]-mephenytoin 4′-hydroxylation, and CYP3A4-mediated midazolam 1′-hydroxylation, with *K*_i_ values of 10.2, 3.7, 5.8, and 12.6 µM, respectively. It weakly inhibited UGT1A1-catalyzed SN-38 glucuronidation, UGT1A6-catalyzed *N*-acetylserotonin glucuronidation, and UGT1A9-catalyzed mycophenolic acid glucuronidation, with IC_50_ values of 131.7, 144.1, and 71.0 µM, respectively. The compound negligibly inhibited CYP1A2, CYP2A6, CYP2B6, CYP2D6, UGT1A3, UGT1A4, and UGT2B7. Aschantin should be examined in terms of potential *in vivo* pharmacokinetic drug interactions attributable to inhibition of CYP2C8, CYP2C9, CYP2C19, and CYP3A4.

## Figures and Tables

**Table 1:** Inhibitory effects of aschantin on cytochrome P450 (CYP) metabolic activities in pooled human liver microsomes.

CYP	Enzyme	IC_50_ (µM)		*k*_inact_ (min^−1^)	*K*_i_ (µM)
		No Pre-incubation	With Pre-incubation *		
1A2	Phenacetin *O*-de-ethylase	>100	>100	-	-
2A6	Coumarin 7-hydroxylase	>100	>100	-	-
2B6	Bupropion hydroxylase	>100	87.9	-	-
2C8	Amodiaquine *N*-de-ethylase	27.8	5.3	0.056	10.2
2C9	Diclofenac 4′-hydroxylase	40.5	14.1	0.044	3.7
2C19	(*S*)-Mephenytoin 4′-hydroxylase	22.7	6.1	0.048	5.8
2D6	Bufuralol 1′-hydroxylase	>100	>100	-	-
3A4	Midazolam 1′-hydroxylase	57.5	14.4	0.062	12.6

* Aschantin was pre-incubated with microsomes for 30 min in the presence of NADPH before adding the substrate. The substrate cocktail concentrations used to measure IC_50_ values were as follows: 50 µM phenacetin, 2.5 µM coumarin, 2.0 µM amodiaquine, 10 µM diclofenac, 100 µM [*S*]-mephenytoin, 5.0 µM bufuralol, and 2.5 µM midazolam. Inhibition of CYP2B6 activity was evaluated separately using 50 µM bupropion. The data represent the averages of three determinations.
